# Microbial Ecology and Metabolism of Emerging Adulthood: Gut Microbiome Insights from a College Freshman Cohort

**DOI:** 10.1080/29933935.2024.2387936

**Published:** 2024-08-19

**Authors:** Alex E. Mohr, Paniz Jasbi, Irene van Woerden, Jinhua Chi, Haiwei Gu, Meg Bruening, Corrie M. Whisner

**Affiliations:** a College of Health Solutions, Arizona State University, Phoenix, AZ, USA; b Center for Health Through Microbiomes, Biodesign Institute, Arizona State University, Tempe, AZ, USA; c Biodesign Center for Personalized Diagnostics, School of Molecular Sciences, Arizona State University, Tempe, AZ, USA; d Community and Public Health, Idaho State University, Pocatello, ID, USA; e Department of Nutritional Sciences, College of Health and Human Development, Pennsylvania State University, University Park, PA, USA

**Keywords:** Microbiome, metabolome, healthy microbiota, state transition, stability, Dirichlet allocation, longitudinal

## Abstract

The human gut microbiome (GM) undergoes dynamic changes throughout life, transitioning from infancy to adulthood. Despite improved understanding over the past years about how genetics, lifestyle, and the external environment impact the GM, limited research has explored the GM’s evolution during late-stage adolescence, especially among college students. This study addresses this gap by investigating the longitudinal dynamics of fecal microbial, functional, and metabolomic signatures in a diverse group of first-year, dormitory-housed college students. A total of 485 stool samples from 246 participants were analyzed, identifying four primary GM community types, predominantly led by *Bacteroides* (66.8% of samples), as well as *Blautia* and *Prevotella*. The *Prevotella*/*Bacteroides* (P/B) ratio emerged as a robust GM composition indicator, predictively associated with 15 metabolites. Notably, higher P/B ratios correlated negatively with p-cresol sulfate and cholesterol sulfate, implying potential health implications, while positively correlating with kynurenic acid. Distinct GM transition and stability patterns were found from a detailed longitudinal subset of 93 participants over an academic year. *Parasutterella* and the *Ruminococcus gnavus group* exhibited positive associations with compositional variability, whereas *Faecalibacterium* and *Eubacterium ventriosum groups* displayed negative associations, the latter suggesting stabilizing roles in the GM. Most notably, nearly half of the longitudinal cohort experienced GM community shifts, emphasizing long-term GM adaptability. Comparing individuals with stable community types to those undergoing transitions, we observed significant differences in microbial composition and diversity, signifying substantial shifts in the microbiota during transitions. Although diet-related variables contributed to some observed variance, diet did not independently predict the probability of switching between community types within the study’s timeframe via multi-state Markov modeling. Furthermore, exploration of stability within dynamic microbiomes among the longitudinal cohort experiencing shifts in community types revealed that microbiome taxa at the genus level exhibited significantly higher total variance than estimated functional and fecal metabolomic features. This suggests tight control of function and metabolism, despite community shifting. Overall, this study highlights the dynamic nature of the late-stage adolescent GM, the role of core taxa, metabolic pathways, the fecal metabolome, and lifestyle and dietary factors, contributing to our understanding of GM assembly and potential health implications during this life phase.

## Introduction

The human gut microbiome (GM), a complex ecosystem of microorganisms residing within the gastrointestinal tract, has emerged as a focal point of research due to its profound impact on health and physiology. Importantly, the GM temporal fluxes are undergoing remarkable transformations over the course of life. While infancy is marked by substantial changes,^1^ the adult microbial community exhibits relative stability but remains susceptible to perturbations that can shift it away from an established community state.^2^ Progressing into mid-to-late adulthood, the GM becomes increasingly unique to individuals.^3^ This phenomenon of individualization is exemplified by a depletion of core genera in the microbiome patterns of healthy aging.^3^ Notably, diet and behavioral factors have been found to impact the GM community and its functional potential,^4^ especially the promotion of butyrate-producing microbes, suggesting these factors as critical targets for enhancing host-microbiome health.^5^ Interestingly, some research highlights a youth-related aging pattern of the GM for long-lived individuals.^6^ Despite this wealth of knowledge, there remains limited understanding of how the human GM shifts throughout adulthood and how these changes influence health outcomes of the host.

The composition of the GM is differentially influenced by a multitude of factors, encompassing host genetics, lifestyle choices, dietary habits, diseases, and interactions with the external environment.^7,8^ Research has underscored the importance of accounting for shared environmental exposures when investigating the GM, as interventions targeting this complex ecosystem must differentiate between true treatment effects and environmental signals.^9^ Indeed, recent work exploring the GM of 8,208 Dutch individuals from a three-generational cohort, comprising 2,756 families, revealed that approximately 6.6% of taxa were heritable, while roughly 48.6% of the taxa exhibited significant associations with cohabitation.^10^ Moreover, even after meticulous adjustment for over 200 host and environmental factors, a substantial portion of the GM variance remained attributable to individual variation. This emphasizes the inherent uniqueness of the GM in each person, where the distinctions between individuals are substantially larger than variations observed within a single individual over time.^11^

Despite this well-recognized variability in community composition among individuals, an understanding of the underlying ecological features guiding GM assembly remains limited.^12^ Recent research has revealed that environmental and host factors explain less than 20% of the variation in microbial composition,^13,14^ emphasizing the substantial roles played by stochastic factors and ecological principles in shaping the GM.^15^ This inherent variation, coupled with individualized stability, underscores the importance of gaining a deeper understanding of the biological underpinnings that can inform microbiome-based preventive and therapeutic strategies. In the context of the GM, distinct community states, such as *Bacteroides*- vs. *Prevotella*-enriched microbiotas, have been identified.^16–18^ However, one underexplored phase of human life is late-stage adolescence, a period when many health behaviors become firmly established. This transitional phase may offer valuable insights into how the GM matures and present potential targets for intervention.

The college experience marks a significant transition for emerging adults, encompassing changes in living arrangements, financial independence, and, crucially, health behaviors, including diet, physical activity, and sleep.^19^ As this diverse population transitions from family-living to independent living conditions, individuals encounter novel challenges while leaving behind familiar ones.^20^ Moreover, the convergence of college students from diverse backgrounds into a shared environment may impose ecological constraints on health behaviors and the GM. Adolescence is characterized by greater interpersonal variation and lower bacterial diversity compared to adulthood, potentially facilitating more pronounced and lasting microbial shifts.^21^ Despite an extensive body of literature demonstrating the GM as a health status indicator, limited research has explored these relationships among college-aged adults. Our prior studies have provided insights into differences in GM composition based on nutritional and behavioral factors.^22–24^ However, to our knowledge, no studies have undertaken an extensive evaluation of GM composition and the fecal metabolome among college students over an entire academic year.

This study addresses these knowledge gaps through a two-fold approach. First, we conducted an extensive assessment of the microbial and metabolomic profiles of the GM in a diverse cohort of emerging adults. Second, we investigated the longitudinal dynamics of this complex ecosystem, shedding light on the stability and shifts in core taxa configurations. Leveraging data collected from 246 first-year college dormitory residents, our analyses provide valuable insights into the GM’s responses to the unique challenges encountered during the initial year of college life. Additionally, our research delves into the emergence of specific microbial communities within this population, tracking the alterations in GM composition over this critical transitional period, evaluating the influence of lifestyle factors on microbiome dynamics within this context, and exploring the potential implications of these GM changes for the metabolic processes occurring in the gastrointestinal tract.

## Materials and methods

### Participants and study design

This research utilizes data gathered from the broader SPARC (Social Impact of Physical Activity and Nutrition in College) study, with detailed methodologies outlined in another source.^25^ Briefly, the SPARC study involved 1,435 individuals from 6 residence halls at a large public university. These participants engaged in online surveys through Qualtrics, and trained research assistants collected their anthropometric measurements at the beginning and end of the academic terms in Fall 2015 and Spring 2016. All individuals gave their written consent, and the study’s procedures received approval from the Institutional Review Board (IRB) at Arizona State University (ASU) under the protocol number 1309009596. The devilWASTE project selected participants from the SPARC study residing in three specific dormitories to provide stool samples. DevilWASTE’s exclusion criteria included being under 18 years old, having certain gastrointestinal conditions (such as malabsorption disorders), eating disorders, recent antibiotic use (within 2–3 months of the study), and conditions that could alter gut microbiota, like HIV, diabetes, or hypertension. Eligibility for inclusion required participation in the SPARC study, fluency in English, and residence in one of ASU’s dormitories.

### Data collection

The devilWASTE study recruited participants during the 2015–2016 academic year, beginning in August 2015. Throughout the year, participants were asked to provide stool samples and information on their anthropometrics, physical activity, and diet at three different points during the SPARC study (the start and end of both fall and spring semesters). Measurements of weight, height, and waist circumference were conducted by skilled research personnel using Seca 869 scales, Seca 217 stadiometers, and Gulick measuring tapes, respectively. These measurements were used to calculate and report the participants” body mass index (BMI) in kg/m.^2^ Lifestyle changes were monitored through questionnaires administered digitally through Qualtrics software, including the Godin-Shephard Leisure-Time Physical Activity Questionnaire for physical activity assessment, which classifies activity levels as vigorous, moderate, or light, followed by summing the total time spent doing moderate-to-vigorous physical acivity.^26^ Depression and anxiety were assessed using questions adapted from validated questionnaires by the American College Health Association-National College Health Assessment II^27^ and Cohen, et al.^28^ respectively. For capturing depression, students were asked ‘How often in the past 1 month have you felt 1) Things were hopeless; 2) Overwhelmed by all you had to do; 3) Very lonely; 4) Very sad; 5) So depressed that it was difficult to function; 6) Overwhelming anxiety? (Response options: never, rarely, sometimes, often)’. For anxiety/stress, students were asked, ‘How often in the past 1 month have you felt: 1) Unable to control the important things in your life?; 2) Confident about your ability to handle your personal problems; 3) Things were going your way; 4) Difficulties were piling up so high that you could not overcome them? (Response options: never, rarely, sometimes, often)’. Dietary habits were self-reported using the National Cancer Institute Dietary Screener Questionnaire,^29^ and alcohol consumption was quantified by weekly drink intake, as reported previously.^24^ The study also estimated average sleep duration by combining self-reported sleep hours during weekdays and weekends. Additionally, academic and psychosocial factors such as participants’ grade point average (GPA), Pell grant status for the fall and spring semesters, time management skills, social interactions, and mental health status were evaluated, with GPA and Pell grant information retrieved from university records. All questionnaire data were analyzed in either continuous (e.g., GPA, age, BMI, dietary data, sleep duration, and physical activity) or categorical format (e.g., race/ethnicity, depression, anxiety, and Pell grant status). Depression and anxiety question data were further dichotomized for analyses as “sometimes/often” vs. “rarely/never”.

### Fecal sample collection and DNA extraction

Research staff delivered fecal sample collection kits to the residence halls of eligible participants. Fecal samples were collected at three timepoints, and participants were asked to report any medication and supplement use within the last 3 months. If participants had taken any antibiotics, antifungals, or probiotics within the previous 3 months, a fecal sample was not obtained. Research staff picked up the fecal samples within 30 min of a participant reported bowel movement and transported them to the laboratory where they were frozen at −80°C until further processing. Frozen samples were thawed at 4°C, and wet weight was recorded to the nearest 0.01 g after subtracting the weight of fecal collection materials. DNA was extracted from approximately 300 mg of feces, collected from the center of the sample, using a modified version of the manufacturer protocol (MoBio Power Soil DNA Isolation Kit #12888–100, MoBio, Carlsbad, CA). Per the manufacturer's recommendations, an initial heating step of 65°C for 10 min was added to the protocol to reduce the influence of inhibitors commonly found in feces and increase DNA yield. DNA concentration and quality were quantified using QIAxpert System (Qiagen, Germantown, MD) according to the manufacturer’s instructions.

### Microbiome sequencing and bioinformatics

High-throughput genomic sequencing of the 16S rRNA gene was performed at the Biodesign Institute at ASU in Tempe, Arizona, using Illumina miSeq technology after ligating 515F and 806 R primers and Illumina adapters via polymerase chain reaction. Negative controls were included and run with the study samples. A detailed report of methods to prepare and sequence DNA has been published.^22,23^ Due to the complex nature of the devilWASTE study design, large number of samples, and low-quality sequencing in some initial runs, three sequencing runs were conducted. In cases where samples were sequenced multiple times, files were merged after performing quality control. This method has been supported by expert census from the Quantitative Insights Into Microbial Ecology 2 (QIIME2) software development team,^30^ the bioinformatic pipeline used in the present analysis. Briefly, paired-end, demultiplexed data were imported and analyzed using QIIME 2 software version 2021.8. To ensure the quality of the sequencing data, raw fastq files were first assessed using FastQC to identify any issues related to base quality, GC content, and sequence duplication levels. The average Phred score across all reads was above 30, indicating high-quality sequencing data. The DADA2 pipeline within QIIME2 was employed to trim low-quality bases (trimming 20 bases from the start of each read) and truncate reads at position 240 to remove low-quality regions. DADA2 was also used for denoising and constructing a feature table using amplicon sequence variants (ASVs). Each sequencing run was processed separately to prevent error modeling. After processing the individual runs, the resulting feature tables and representative sequences were merged using QIIME2’s “feature-table merge” and “feature-table merge-seqs” functions. This merging process pools all features from duplicated samples into one and summarizes all identical features, ensuring that data from multiple sequencing runs are combined accurately without losing any information. This step is crucial for integrating data from multiple runs to create a comprehensive dataset that includes all high-quality reads. The final dataset included a total of 22,628,375 high-quality reads, with median reads per sample of 46,238 (run 1), 33395 (run 2), and 77,808 (run 3). The quality of the final fastq files was high, with over 90% of reads passing initial quality control filters. Scripts and batch files used for the quality control process have been included in this study’s GutHub repository (see “Availability of data and material”) to enhance the reproducibility of our methods.

The ASV feature table was processed through the feature-classifier plugin, pre-trained to the SILVA rRNA database (138.1; 99% ASVs from 515F/806 R region of sequences).^31^ Following this, a phylogenetic tree was created with the help of the fragment-insertion plugin, using SILVA. This was done at a predefined rarefaction threshold for p-sampling depth, aiming to include high-quality reads and adjust for varying sequencing depths across samples. Sequences classified as Archaea, unclassified at the Phyla-level Bacteria, as well as mitochondrial and plant DNA, were excluded. Additionally, ASVs that were singletons in all samples were discarded. To evaluate alpha-diversity, high-quality reads were inferred, and the sample sequencing depth was normalized using a rarefaction threshold of 7,600 before calculating the Shannon diversity index.^32^ Beta-diversity was assessed using the Bray-Curtis dissimilarity metric. Estimated functional potential of the overall bacterial community was surveyed via the Phylogenetic Investigation of Communities by Reconstruction of Unobserved States 2 (PICRUSt2) algorithm (v2.4.2).^33^ Pathway abundances were inferred based on structured pathway mappings of Enzyme Commission gene families to the MetaCyc database.^34^ Subsequently, a “phyloseq” object (v1.38.0.) was generated for further analyses and visualizations in R (v4.1.2.).

Classification of GM community clustering was established using Dirichlet multinomial mixture (DMM) modeling^35^ for the entire set of 485 samples at the genus level using the R package “DirichletMultinomial” (v1.42.0). Only core taxa prevalent in at least 30% of samples with a minimum count of 10 were considered and entered into the model. Goodness-of-fit was assessed via Laplace minima. We assessed latent structures within the GM using the Potential of Heat-diffusion for Affinity-based Trajectory Embedding (PHATE) algorithm (phateR v1.0.7) with default parameters at the genus level. Briefly, PHATE is a visualization method that captures both local and global nonlinear structure using an information-geometric distance between datapoints.^36^ It does not impose any strong assumptions on the structure of the data, which better preserves patterns in data, including clusters and branching.^37^

### Fecal metabolomics

Acetonitrile (ACN), methanol (MeOH), ammonium acetate, and acetic acid, all liquid chromatography-mass spectrometry (LC-MS) grade, were purchased from Fisher Scientific (Pittsburgh, PA). Ammonium hydroxide was bought from Sigma-Aldrich (Saint Louis, MO). DI water was provided in-house by a Water Purification System from EMD Millipore (Billerica, MA). PBS was bought from GE Healthcare Life Sciences (Logan, UT). The standard compounds corresponding to the measured metabolites were purchased from Sigma-Aldrich (Saint Louis, MO) and Fisher Scientific (Pittsburgh, PA).

For sample preparation, each fecal sample (~20 mg) was homogenized in 200 µL MeOH:PBS (4:1, v:v, containing 1,810.5 μM^13^C_3_-lactate and 142 μM^13^C_5_-glutamic acid) in an Eppendorf tube using a Bullet Blender homogenizer (Next Advance, Averill Park, NY). Then 800 µL MeOH:PBS (4:1, v:v, containing 1,810.5 μM^13^C_3_-lactate and 142 μM^13^C_5_-glutamic acid) was added, and after vortexing for 10 s, the samples were stored at −20°C for 30 min. The samples were then sonicated in an ice bath for 30 min. The samples were centrifuged at 14,000 RPM for 10 min (4 °C), and 800 µL supernatant was transferred to a new Eppendorf tube. The samples were then dried under vacuum using a CentriVap Concentrator (Labconco, Fort Scott, KS). Prior to the MS analysis, the obtained residue was reconstituted in 150 μL 40% PBS/60% ACN. A quality control (QC) sample was pooled from all the study samples.

The untargeted LC-MS metabolomics method used here was modeled after that developed and used in a growing number of studies.^22,23^ Briefly, all LC-MS experiments were performed on a Thermo Vanquish UPLC-Exploris 240 Orbitrap MS instrument (Waltham, MA). Each sample was injected twice with 10 µL for analysis using negative ionization mode and 4 µL for analysis using positive ionization mode. Both chromatographic separations were performed in hydrophilic interaction chromatography (HILIC) mode on a Waters XBridge BEH Amide column (150 × 2.1 mm, 2.5 µm particle size, Waters Corporation, Milford, MA). The flow rate was 0.3 mL/min, auto-sampler temperature was kept at 4 °C, and the column compartment was set at 40 °C. The mobile phase was composed of Solvents A (10 mM ammonium acetate, 10 mM ammonium hydroxide in 95% H_2_O/5% ACN) and B (10 mM ammonium acetate, 10 mM ammonium hydroxide in 95% ACN/5% H_2_O). After the initial 1 min isocratic elution of 90% B, the percentage of Solvent B decreased to 40% at *t* = 11 min. The composition of Solvent B was maintained at 40% for 4 min (*t* = 15 min), and then the percentage of B gradually went back to 90% to prepare for the next injection. Using a mass spectrometer equipped with an electrospray ionization (ESI) source, we collected untargeted data from 70 to 1050 m/z.

To identify peaks from the MS spectra, we made extensive use of the in-house chemical standards (~600 aqueous metabolites), and in addition, we searched the resulting MS spectra against the HMDB library, Lipidmap database, METLIN database, as well as commercial databases including mzCloud, Metabolika, and ChemSpider. The absolute intensity threshold for the MS data extraction was 1,000, and the mass accuracy limit was set to 5 ppm. Identifications and annotations used available data for retention time (RT), exact mass (MS), MS/MS fragmentation pattern, and isotopic pattern. We used the Thermo Compound Discoverer 3.3 software for aqueous metabolomics data processing. The untargeted data were processed by the software for peak picking, alignment, and normalization. To improve rigor, only the signals/peaks with CV < 20% across QC pools, and the signals showing up in >80% of all the samples were included for further analysis. A Canberra distance matrix was computed on the metabolomics feature table, as previously described.^38^

### Statistical analysis

All statistical analyses were conducted using R statistical software (version 4.3.1, R Core Team, 2023). Anthropometric, behavioral, and dietary data from participants was first assessed for normality using QQ-plots and Shapiro-Wilk’s test. Log transformations were performed where appropriate. Associations between these factors were assessed with Spearman’s rank correlation method. Differences between males and females were assessed with t-tests or Mann–Whitney U tests, depending on normality.

For the overall assessment of the GM (*n* = 485), alpha diversity was assessed for normality (Shapiro-Wilk’s tests) and log-transformed. Differences between community-type categories were analyzed by a linear mixed-effects model using the “nlme” package (v3.1–163), assessing alpha diversity across the partitions, controlling for sex, race/ethnicity, BMI, and time of collection, using individuals as a random effect. Pairwise comparisons were assessed using the Tukey method. Assessing beta diversity, a permutational multivariate analysis of variance (PERMANOVA) test was constructed for Bray-Curtis dissimilarities in the “vegan” package (v2.6–4) testing the effects of the individual (nested factor), DMM assignment, sex, race/ethnicity, BMI, and time of sample collection (number of permutations = 999). Next, log10-transformed ratios were formed for *Prevotella*/*Bacteroides* and *Blautia*/*Bacteroides* and entered into exploratory multiple regression models with dietary, behavioral, and anthropometric covariate data. Multiple regression models were also used to assess the relationship between these ratios, and all reliably detected fecal metabolites (Pareto-scaled and log10-transformed), controlling for sex, race/ethnicity, BMI, and time of sample collection.

Longitudinal samples (participant *n* = 93; sample *n* = 279) were additionally assessed to better capture temporal associations across the three time points within an academic year. The overall inter-individual and intra-individual Bray-Curtis dissimilarities of the longitudinal sample set were assessed by a Mann-Whitney U test. Linear mixed-effects models were then used to explore factors influencing individual-level changes in the GM. These models utilized sqrt-transformed median Bray-Curtis dissimilarity between each time point pair (1 to 2, 1 to 3, and 2 to 3) as the dependent variable. Independent variables included dietary, behavioral, and anthropometric covariate data. Participant identity was treated as a random effect in the models, which were executed using the “nlme” package. In addition, mean Shannon index values and centered log-ratio (CLR) transformed (after adding a pseudocount of 1) mean values of core genera, detected in at least 30% of samples with a minimum count of 10, over the three time points, were regressed against the sqrt-transformed median intra-individual Bray-Curtis dissimilarity, with beta coefficients representing effect sizes. Changes in intra-individual sqrt-transformed median Bray-Curtis dissimilarity, Shannon index, and relative abundance of significant genera from prior regression analyses were correlated against shifts in the CLR abundance of core estimated functional MetaCyc pathways (identified in at least 30% of samples with a minimum count of 10). These pathways were inferred using the PICRUSt2 algorithm, and correlations were determined using Spearman’s rank correlation method.

We categorized longitudinal samples based on their stability within initially assigned DMM community clusters (i.e., Bacteroides-1 [Bact1], Bacteroides-2 [Bact2], Blautia [Blau], or Prevotella [Prev]) or their transition to a different state at subsequent time points. The impact of community-type stability and transitions on microbial diversity and composition was evaluated using Kruskal–Wallis tests, comparing intra-individual Bray-Curtis dissimilarities, Shannon index values, and the relative abundance of genera linked to community variability. For post-hoc analysis, we employed Dunn’s test to perform pairwise comparisons between groups.

A Chi square test goodness-of-fit with 16 categories (i.e., Bact1 to Bact1, Bact1 to Bact2, Bact1 to Blau, Bact1 to Prev, Bact2 to Bact1, Bact2 to Bact2, Bact2 to Blau, Bact2 to Prev, Blau to Bact1, Blau to Bact2, Blau to Blau, Blau to Prev, Prev to Bact1, Prev to Bact2, Prev to Blau, and Prev to Prev) corresponding to all possible transitions (including stability within a community type across time points) was used to determine whether the observed transitions between community type followed the expected distribution based on their prevalence within the cohort. The expected probability of transition from community type A to B (A-to-B) was calculated using the formula:

$$P\left(A\to B\right)=\frac{\mathrm{Number}\;\mathrm{of}\;\mathrm{possible}\;A-\mathrm{to}-B\;\mathrm{transitions}\;\mathrm{within}\;\mathrm{the}\;\mathrm{dataset}}{\mathrm{Total}\;\mathrm{number}\;\mathrm{of}\;\mathrm{possible}\;\mathrm{transitions}\;\mathrm{within}\;\mathrm{the}\;\mathrm{dataset}}$$

This approach accounted for the unequal distribution of samples across the community types. The expected frequency for each A-to-B transition was then calculated by multiplying the total number of transitions by the expected probability of that transition. The observed number of transitions for each category was tallied based on the longitudinal data. Standardized residuals were computed to evaluate the discrepancy between observed and expected transition rates, with a value beyond ±1.96, indicating a significant deviation at the 0.05 level.

To quantify the effects of multiple covariates on community-type distribution, we performed a distance-based redundancy analysis (db-RDA) using the “vegan” package. This method facilitates the understanding of how much variance in community composition can be explained by the measured covariates. The db-RDA was conducted on a Bray-Curtis dissimilarity matrix derived from the longitudinal community-type data, with covariates such as dietary habits and lifestyle factors included as explanatory variables. Permutation tests (*n* = 999permutations) were used to assess the significance of the covariates in explaining the variation in community-type composition. For the analysis of transition probabilities between community types over time, we employed multi-state Markov (MSM) models using the “msm” package (v1.7). This approach models the probabilities of transitioning from one state to another over discrete time intervals, considering the longitudinal nature of the data. We defined the transition model with community type as states and days as time information. Transition intensities and the impact of covariates were estimated, with significance tested using a log-likelihood ratio test (function: *lrtest.msm*). Hazard ratios and 95% confidence intervals for each covariate’s impact on transitions were calculated (function: *hazard.msm*).

To explore the variability in microbial communities within and between individuals that shifted community type, we calculated intra-individual and inter-individual variance, along with the Intraclass Correlation Coefficients (ICC) of genera and estimated function (using a lower feature threshold: i.e., identified in at least 10% of samples with a minimum count of 10) and the fecal metabolome. The calculation involved using a repeated-measures analysis of variance framework, where the individual subjects were treated as random effects, and the microbial taxa abundances were the repeated measures. Specifically, variance components were estimated using the *lmer* function from the “lme4” package, and the ICCs were calculated as:

$$\mathrm{ICC}=\frac{\mathrm{Inter}\;-\;\mathrm{Individual}\;\mathrm{Variance}}{\mathrm{Inter}\;-\;\mathrm{Individual}\;\mathrm{Variance}\;+\;\mathrm{Intra}\;-\;\mathrm{Individual}\;\mathrm{Variance}}$$

Kruskal–Wallis tests were used to compare total variance (inter-individual + intra-individual variance), intra-individual variance, and ICC across genera, estimated functional pathways, and the fecal metabolome with Dunn’s test for pairwise comparisons. Associations between CLR abundance and intra-individual variance were calculated for taxa, estimated functional pathways, and metabolites separately with Spearman’s rank correlation method. For all statistical tests, a *P*-value of 0.05 was used to denote statistical significance. In each instance where multiple hypotheses were tested, the type I error was controlled by using the Bonferroni correction (*P*_adj_ < 0.05).

## Results

### Profiling the gut microbiome of college students reveals distinct clustering of microbial communities and metabolite expression

**Figure 1.**
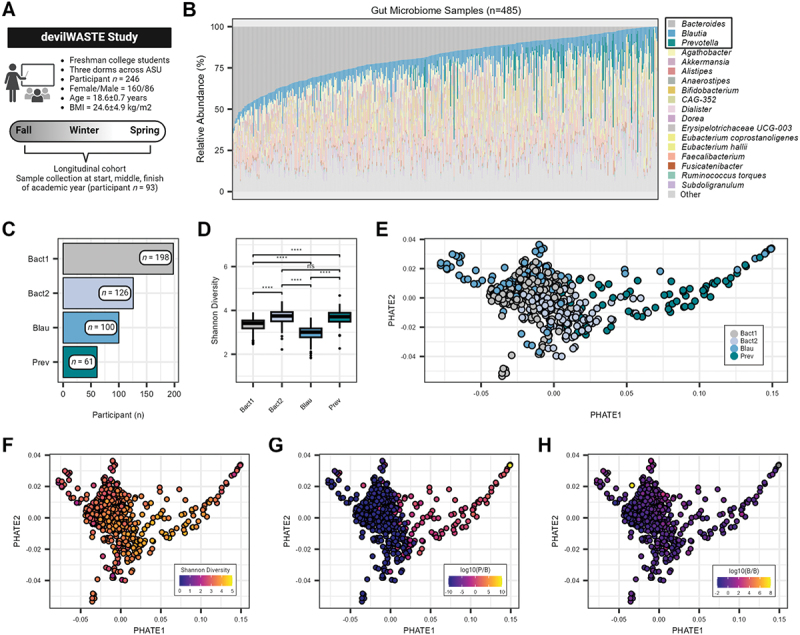
Overview of study design, microbial taxa distribution, community types, diversity, and potential of heat-diffusion for affinity-based trajectory embedding (PHATE) visualization. (A) outline of the devilWASTE study conducted over a full academic year at Arizona state university. (B) relative abundance distribution of bacterial taxa in all analyzed samples (*n* = 485), highlighting taxa accounting for ≥1.0% relative abundance; remaining taxa grouped as “other.” *Bacteroides* is displayed in descending order and highlighted alongside the other relevant community type driving taxa, *Blautia* and *Prevotella*. (C) distribution of samples across four community types identified through Dirichlet multinomial mixture (DMM) modeling. (D) Shannon diversity comparisons across the four community types, presented as boxplots with median, 1st, and 3rd quartiles, and whiskers extending 1.5× the interquartile range; (**** indicates pairwise comparisons with *P*_adj_ ≤ 0.001). (E-H) visualization of samples using the PHATE algorithm scatter plots, color-coded by (E) community type, (F) Shannon diversity, (G) log10-transformed *Prevotella*/*Bacteroides* ratio (P/B), and (H) log10-transformed *Blautia*/*Bacteroides* ratio (B/B).

We conducted a comprehensive analysis of 485 whole stool samples, consisting of 104 single-time-point and 381 multiple-time-point (102 two-time-point and 279 three-time-point) collections obtained from 246 healthy college students during their first year of dormitory living (Figure 1A). Among these, a subset of 93 participants provided complete longitudinal sample sets over the academic year, allowing us to examine the dynamics of the fecal microbiome and metabolism in late-stage adolescents. We collected health and behavior data, including demographic, anthropometric, dietary, activity, and sleep information (Supplemental Table S1). Notably, differences between females and males were detected in anthropometric and dietary data (t-tests, *p* ≤ 0.028), but age, BMI, depression, anxiety, average sleep duration, and weekly alcohol intake showed no significant differences (*p* ≥ 0.149). The mean grade point average (GPA) was 3.07 ± 0.73, with no gender-based variation (t-test, *p* = 0.164). Approximately one-third of participants received the Pell grant (*n* = 83), highlighting the inclusion of students from under-resourced backgrounds within the cohort. Importantly, dietary fiber intake for both males (*n* = 86; 19.02 ± 5.59 g/day) and females (*n* = 160; 14.82 ± 3.39 g/day) fell below the recommended Adequate Intake levels per the Dietary Guidelines for Americans (males: 38 g/day; females: 25–26 g/day),^39^ suggesting potential dietary challenges (Supplemental Table S1).

Assessing the taxonomic composition of all microbiome samples, we noted substantial coverage of *Bacteroides* at the genus level (mean relative abundance: 18.56%; Figure 1B). Other notable contributions included *Blautia* (8.95%), *Faecalibacterium* (8.65%), *Bifidobacterium* (7.13%), and to a lesser extent, *Prevotella* (2.86%). Based on the apparent gradient, we first sought to classify ecological states of the GM of the entire data set of participants via DMM modeling. Using Laplace’s index for establishing model fit, we found four clusterings of microbial communities, with the main community driver being *Bacteroides*, accounting for 66.8% of samples and comprised of types 1 (Bact1: *n* = 198) and 2 (Bact2: *n* = 126), *Blautia* for type 3 (Blau: *n* = 100), and *Prevotella* for type 4 (Prev: *n* = 61; Figure 1C; Supplemental Figure S1). Two types of *Bacteroides* profiles have been previously reported, with Bact2 being implicated with a disease-associated microbial configuration.^40–43^ Assessing alpha diversity across the partitions, controlling for sex, race/ethnicity, BMI, and time of collection, revealed significant differences between nearly all four classifications (pairwise comparison tests, *P*_adj_ ≤ 2.05e-08; Figure 1D), save for Bact2 vs. Prev (*P*_adj_ = 0.906). Exploratory analysis using PERMANOVA indicated that DMM classification explained a significant portion of the model variance (*R*^2^ = 0.075, *p* < 0.001), although the individual remained the primary source of dissimilarity (*R*^2^ = 0.645, *p* < 0.001; Supplemental Table S2). This result aligns with previous research highlighting the importance of community composition in driving microbial community structure.^44,45^ Other factors, such as sex, race/ethnicity, BMI, and time of collection (beginning, middle, and end of the academic year) were also significant, though explained comparatively low amounts of variance (*R*^2^ ≤ 0.012, *p* < 0.001).

To elucidate potential differences among GM community structures, we implemented the PHATE algorithm to better delineate latent structures within the entire devilWASTE cohort. PHATE algorithm analysis revealed distinct branching patterns, with notable clustering of Prev and Blau community types (Figure 1E). Concurrently, Shannon diversity mapping revealed elevated values near the origins of the Prev branch and the Bact2 cluster (Figure 1F), suggesting that the *Prev* and *Bact2* communities may encompass a more diverse array of microbial features. This heightened diversity could significantly influence the community’s functional potential and its resilience to perturbations. Furthermore, our findings suggest that the *Prevotella/Bacteroides* (P/B) ratio could serve as an indicator of an individual’s GM composition (Figure 1G). This observation aligns with prior research linking the P/B ratio to dietary patterns and associated health implications.^16–18^ In contrast, the *Blautia/Bacteroides* (B/B) ratio did not exhibit a similar trend (Figure 1H).

Based on the apparent branching from the DMM model and the P/B ratio, we next investigated potential dietary, behavioral, and anthropometric factors that may be predictive of P/B ratio. Using linear modeling, we found that the DMM assignment most strongly predicted P/B ratio, explaining 48.85% of the model variance (*P*_adj_ = 3.4e-10). Other significant factors included dormitory (explained variance = 2.20%, *P*_adj_ = 0.006), GPA (explained variance = 2.98%, *P*_adj_ = 0.001), and number of academic credits (explained variance = 1.62%, *P*_adj_ = 0.039). Notably, demographic, anthropometric, physical activity, behavioral (depression and anxiety), and dietary factors showed little association and significant effects with P/B ratio (Supplemental Table S3). The information on dietary habits captured by our questionnaires was limited, hence these results need to be interpreted with caution. Assessing the B/B ratio revealed only the DMM assignment as a significant predictor, after *P*-value correction (explained variance = 13.83%, *P*_adj_ = 6.77e-08; Supplemental Table S4).

**Figure 2.**
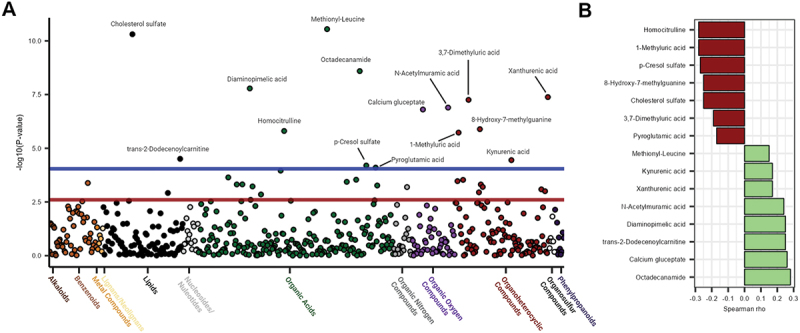
Metabolic signatures associated with *Prevotella/Bacteroides* ratio and correlation analysis. (A) fecal metabolites, categorized by super-family, with corresponding -log10 p-values derived from regression models predicting *Prevotella*/*Bacteroides* ratio while adjusting for sex, BMI, and time. Metabolites above the red line were deemed significant after multiple-hypothesis correction (*P*_adj_ < 0.05), while the blue line represents the unadjusted *P*-value threshold. (B) spearman correlation coefficients illustrating the relationships between each metabolite and the *Prevotella*/*Bacteroides* ratio, after controlling for covariates and applying multiple-hypothesis correction (*P*_adj_ < 0.05).

To detect a potential metabolic signature of the P/B ratio, we performed regression analysis against all reliably detected metabolites (*n* = 552) from fecal samples in the devilWASTE study, controlling for sex, race/ethnicity, BMI, and time of collection. There were 15 features that were significantly associated with the P/B ratio after *P*-value correction (*P*_adj_ < 0.05; Figure 2A). These metabolites spanned four super classes, including lipids and lipid-like molecules (cholesterol sulfate and trans-2-dodecenoylcarnitine), organic acids and derivatives (methionyl-leucine, octadecanamide, diaminopimelic acid, homocitrulline, p-cresol sulfate, and pyroglutamic acid), organic oxygen compounds (n-acetylmuramic acid and calcium gluceptate), and organoheterocyclic compounds (xanthurenic acid, 3,7-dimethyluric acid, 8-hydroxy-7-methylguanine, 1-methyluric acid, and kynurenic acid). The strongest positive correlations included octadecanamide, calcium gluceptate, trans-2-dodecenoylcarnitine, and diaminopimelic acid (Spearman’s *rho* ≥0.25; Figure 2B), whereas the strongest negative correlations included homocitrulline, 1-methyluric acid, p-cresol sulfate, 8-hydroxy-7-methylguanine, and cholesterol sulfate (Spearman’s *rho* ≤ −0.25). Of particular interest was p-cresol sulfate, a microbial-generated protein-bound uremic toxin associated with kidney disease progression,^46^ was among the identified metabolites. Kynurenic acid, a metabolite known for its potential neuroprotective properties^47^ and anti-inflammatory activity^48^ is produced during the breakdown of tryptophan primarily within the host’s metabolic pathways.

Performing the same analysis on the B/B ratio, we detected six features that were significant (*P*_adj_ < 0.05; Supplemental Figure S2A). These included metabolites mostly from the super class, organic acids, and derivatives, such as p-cresol sulfate, allysine, levetiracetam, L-gamma-glutamyl-L-leucine, and N-Acetyl-L-aspartic acid. Others were from lipids and lipid-like molecules, lithocholic acid (a secondary bile acid that acts as a detergent to solubilize fats for absorption and is itself absorbed), and organoheterocyclic compounds, dehydroascorbic acid (an oxidized form of ascorbic acid). In comparison to the P/B ratio, the correlations were small (Spearman’s *rho* |<| 0.25; Supplemental Figure S2B), though p-cresol sulfate was negatively correlated (Spearman’s *rho* = −0.14). Others, such as allysine (Spearman’s *rho* = 0.14) and dehydroascorbic acid (Spearman’s *rho* = 0.02) showed a positive correlation, whereas L-gamma-glutamyl-L-leucine showed a negative correlation (Spearman’s *rho* = −0.08).

### Longitudinal dynamics and keystone taxa contributions over the academic year

**Figure 3.**
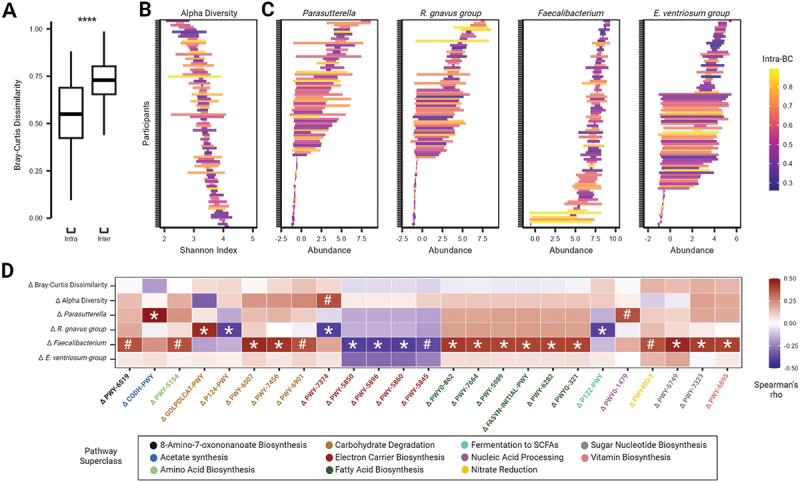
Longitudinal gut microbiome dynamics and functional correlations. (A) boxplots comparing intra- and inter-individual Bray-Curtis dissimilarities, with median values represented by the central line, quartiles by the box, and range (1.5× the interquartile range) by the whiskers (Mann-Whitney U test, ****p < 2.2e − 16). (B-C) histograms showcasing the distribution of alpha diversity (B) and relative abundance of key genera (C) associated with intra-individual bray-Curtis dissimilarity. Bars represent the range of genus centered log ratio relative abundance across three sample points for each individual, with color gradation indicating the level of intra-individual Bray-Curtis dissimilarity. (D) heatmap illustrating Spearman correlations between variations in inter-individual Bray-Curtis dissimilarity, alpha diversity, and significant genus relative abundances with variations in estimated microbial functional pathways. Pathways are color-coded by their superclass. The heatmap displays correlation results with statistically significant adjusted *P*-values (^#^*P*_adj_ < 0.1; *P_adj_ < 0.05).

Among the 246 study participants, a subset of 93 individuals provided complete sample sets at the onset, midpoint, and conclusion of the academic year. This subset facilitated a detailed exploration of the longitudinal dynamics within distinct GM communities. We observed that the inter-individual GM variability was markedly higher than the intra-individual variability (Mann-Whitney U test, *p* ≤ 2.20e − 16; Figure 3A). This suggests distinct, individualized microbial signatures that are relatively stable over time. To decipher the factors contributing to intra-individual GM changes, we employed linear mixed-effects (LME) modeling. These models leveraged participant-specific predictors, age, sex, sleep patterns, depression, anxiety, GPA, anthropometric measurements, and dietary data. The analysis did not reveal significant influences of these factors on intra-individual Bray-Curtis dissimilarity (LME, *p* ≥ 0.179). This suggests that while these variables may play a role in the overall GM composition, they do not significantly account for changes within an individual’s microbiome over time. In addition, our investigation found that the intra-individual Bray-Curtis dissimilarity was not significantly associated with alterations in alpha diversity (LME, *p* = 0.233; Figure 3B). This observation indicates that while the overall community composition might exhibit fluctuations, the underlying richness and evenness of the microbiome remained relatively unchanged over the course of one academic year for the participants. This underscores resilience in microbial community diversity, where the core attributes of the microbiome remain relatively stable even in the face of compositional shifts.

Focusing on core genera (*see* Methods), we found significant relationships with certain taxa (Figure 3C). *Parasutterella*, known for its reliance on amino acids for metabolic activities,^49^ displayed a positive association with intra-individual Bray-Curtis dissimilarity (LME, effect size = 5.91, *p* = 0.018). *Ruminococcus gnavus group*, a known mucin glycan forager,^50^ also exhibited a positive association (effect size = 5.59, *p* = 0.043). In comparison, genera associated with butyrate production, like *Faecalibacterium* and *Eubacterium ventriosum group*, were negatively associated with compositional intra-individual variability, suggesting their potential role in stabilizing the GM (effect size = −3.49 and −4.52, respectively, *p* ≤ 0.027).

Delving deeper into the functional implications of these associations, we correlated changes in these key taxa with shifts in core-estimated microbial functions (MetaCyc pathways, *n* = 295). Notably, variations in *Parasutterella* positively correlated with changes in the reductive acetyl coenzyme A pathway I (CODH-PWY), involved in acetate formation from acetyl-CoA (Spearman’s *rho* = 0.49; *P*_adj_ = 3.0e-04; Figure 3D). Variations in the *R. gnavus group* showed a complex relationship with multiple pathways, indicating its diverse metabolic capabilities. It positively correlated with the superpathway of glycerol degradation to 1,3-propanediol (GOLPDLCAT-PWY; Spearman’s *rho* = 0.45, *P*_adj_ = 0.002) and negatively with pathways involved in glucose fermentation to lactate (Bifidobacterium shunt: P124-PWY; Heterolactic fermentation: P122-PWY) and menaquinone (vitamin K2) biosynthesis (1,4-dihydroxy-6-naphthoate biosynthesis I: PWY-7374) (Spearman’s *rho* ≤ −0.39, *P*_adj_ ≤ 0.042).

In comparison, *Faecalibacterium* had distinct correlational patterns with notable positive associations with pathways under the carbohydrate degradation, fatty acid biosynthesis, and sugar nucleotide biosynthesis superclasses. The carbohydrate degradation pathways, 4-deoxy-L-threo-hex-4-enopyranuronate degradation (PWY-6507; Spearman’s *rho* = 0.45; *P*_adj_ = 0.003) and β-(1,4)-mannan degradation (PWY-7456; Spearman’s *rho* = 0.43; *P*_adj_ = 0.007) act on complex polymers. Positive correlations relating to fatty acid biosynthesis included the superpathway of fatty acid biosynthesis initiation (FASYN-INITIAL-PWY), (5Z)-dodecenoate biosynthesis I (PWY0–862), oleate biosynthesis IV (PWY-7664), palmitoleate biosynthesis I (PWY-6282), stearate biosynthesis II (PWY-5989), and mycolate biosynthesis (PWYG-321; Spearman’s *rho* ≥0.40; *P*_adj_ ≤ 0.027). Three pathways within the electron carrier biosynthesis superclass (for menaquinones) were negatively correlated with *Faecalibacterium*, including the superpathways of menaquinol-6 biosynthesis (PWY-5850), menaquinol-10 biosynthesis (PWY-5896), and demethylmenaquinol-6 biosynthesis I (PWY-5860; Spearman’s *rho* ≤ −0.41; *P*_adj_ ≤ 0.029). These findings suggest *Faecalibacterium*‘s role in maintaining gut homeostasis through diverse metabolic activities. Intriguingly, no significant correlations emerged for the Bray-Curtis dissimilarity, alpha diversity, or *E. ventriosum group* (*P*_adj_ ≥ 0.085).

### Gut microbiome community type dynamics and transition analysis over an academic year

**Figure 4.**
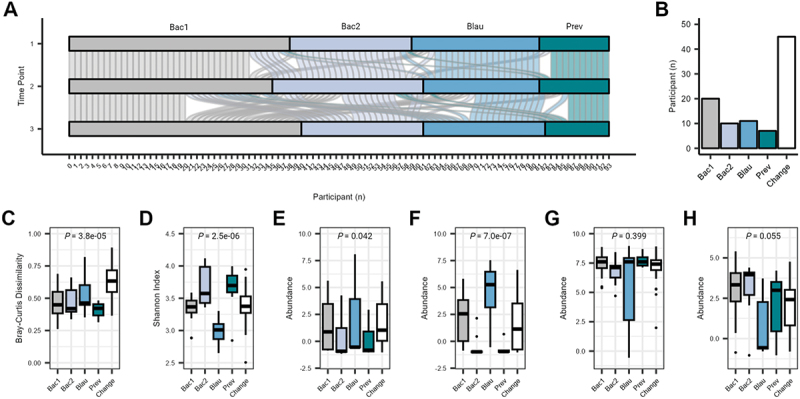
Longitudinal dynamics of gut microbiome community type among college students. (A) individual transitions in community types as determined by Dirichlet multinomial mixture (DMM) modeling over the academic year, represented by community clusters: Bact1 (Bacteroides-1), Bact2 (Bacteroides-2), Blau (Blautia), and prev (Prevotella). (B) distribution of the study cohort across time by DMM classification, illustrating community type stability or shift. (C) boxplot of intra-individual Bray-Curtis dissimilarity scores depicting compositional variability within gut microbiome over time. (D) boxplot of Shannon diversity index values indicating intra-individual alpha diversity. (E-H) boxplots of centered log-ratio (clr) transformed abundances of (E) *Parasutterella*, (F) *Ruminococcus gnavus group*, (G) *Faecalibacterium*, and (H) *Eubacterium ventriosum* correlated with community type stability, analyzed using Kruskal-Wallis tests to detect significant differences across DMM classifications. Boxplot elements denote the median (central line) and interquartile range (box), while the whiskers represent the range within 1.5 times the interquartile distance from the box edges. Outliers are depicted as individual points beyond the whiskers.

The microbial composition of our longitudinal cohort (*n* = 93) fell into distinct DMM community clusters: 38 were classified as Bact1, 21 as Bact2, 22 as Blau, and 12 as Prev dominate, at baseline (Figure 4A). The dynamic nature of the GM was evident as nearly half of the participants (*n* = 45) experienced shifts in their community state throughout the academic year, suggesting permissivity between DMM classifications (Figure 4B). Comparing participants with stable community types to those who shifted, statistically significant variations were observed in within-subject microbial composition, measured by Bray-Curtis dissimilarities, and in microbial diversity, quantified using the Shannon index (Kruskal–Wallis test, *p* ≤ 3.8e-05; Figures 4C–D). More specifically, individuals transitioning between community types exhibited markedly higher microbial variability compared to those maintaining a Bact1, Bact2, or Prev state (Dunn’s test, *P*_adj_ ≤ 0.034). For alpha diversity, the Blau dominated community type stood out for its significantly lower Shannon diversity index relative to the other community classifications (*P*_adj_ ≤ 0.022), suggesting a more homogenous microbial composition within this group.

At the genus level, we revisited previously identified microbial markers associated with intra-individual community variability (Figure 3C). Significant distinctions were noted for *Parasutterella* and the *R. gnavus group* (*p* ≤ 0.042; Figures 4E–F). Conversely, no significant differences were detected for *Faecalibacterium* and the *E. ventriosum group* (*p* ≥ 0.055; Figures 4G–H). While a trend was observed, indicating a higher relative abundance of *Parasutterella* in participants with shifting community types compared to those with stable Bact1 and Prev types, these findings did not reach statistical significance after Bonferroni multiple comparison correction (*P*_adj_ ≤ 0.131). Notably, the *R. gnavus group* was found in significantly lower abundances in individuals with Bact1 and Prev community types when contrasted with shifts to other community types (*P*_adj_ ≤ 0.015).

**Figure 5.**
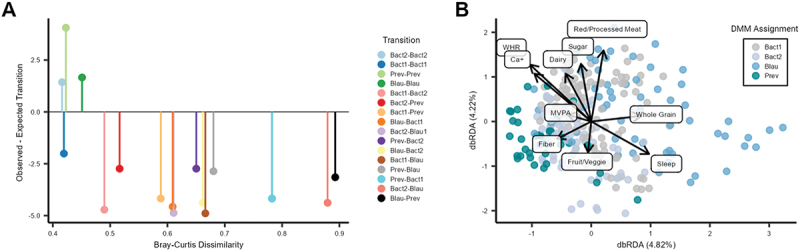
Analysis of community type transitions and contributing factors. (A) visualization of observed versus expected frequencies for the 16 potential community type transitions across the academic year, based on Dirichlet multinomial mixture (DMM) model classifications: Bact1 (Bacteroides-1), Bact2 (Bacteroides-2), Blau (Blautia), and prev (Prevotella). The y-axis displays the standardized residuals from the Chi Square goodness of fit test, quantifying the deviation of observed transitions from expected values. The x-axis shows the median Bray-Curtis dissimilarity, reflecting the magnitude of compositional changes associated with each transition. (B) scatterplot from distance-based redundancy analysis (dbRDA) on the Bray-Curtis dissimilarity matrix, incorporating dietary, anthropometric, and behavioral predictors to elucidate their relative contributions to gut microbiome variation over time. Each point represents an individual sample, color-coded according to dmm-defined community type, highlighting the microbiome’s response to environmental and lifestyle influences.

We quantified the frequency of transitions between the 16 possible states and observed a low incidence of shifts among the *Bacteroides*, *Blautia*, and *Prevotella* dominated communities (Figure 4A; Supplemental Table S5). This observation prompted an investigation into the extent of compositional changes required for transitions, such as from Bact1 to Prev, which are hypothesized to necessitate substantial microbial shifts based on existing literature.^40^ Intra-community transitions for Bact2 to Bact2, Prev to Prev, and Blau to Blau were found to occur more frequently than expected under a model of random switching (chi-square test, *p* = 2.2e-16), suggesting a tendency toward community stability within certain types (Figure 5A). Notably, transitions within the same community type were associated with a lower community dissimilarity (Bray-Curtis dissimilarity <0.45). In contrast, transitions between divergent community types, such as Prev to Bact1, Bact2 to Blau, and Blau to Prev, were characterized by higher dissimilarity measures (Bray-Curtis dissimilarity > 0.75), indicating significant compositional alterations (Kruskal–Wallis test, *p* = 4.1e-05; Figure 5A: y-axis).

Subsequent analyses were conducted to elucidate the influence of dietary, anthropometric, and behavioral factors on gut microbiota community shifts over the study duration (Figure 5B). Variability attributable to individual participants was found to be the predominant source of variation in community composition (dbRDA on Bray-Curtis dissimilarity; adjusted *R*^2^ = 0.75, *p* < 0.001). Diet-related variables such as red/processed meat consumption (adjusted *R*^2^ = 0.05, *p* = 0.007), dietary fiber intake (adjusted *R*^2^ = 0.01, *p* = 0.010), and fruit/vegetable consumption (adjusted *R*^2^ = 0.02, *p* = 0.030) contributed to a smaller, yet significant proportion of the observed variance. Of note, sex had a non-significant bearing when added to model (*R*^2^ < 0.01, *p* = 0.919). To predict the probabilities of community-type transitions over time, we employed a multi-state Markov model incorporating the aforementioned covariates. However, when evaluated independently, these covariates did not show a significant association with the probability of switching between community types (Log-likelihood ratio test, *p* > 0.05). This suggests that while individual behavioral and dietary patterns did influence GM composition, they did not singularly determine community-type transitions within the time frame of our study.

### Intra- and inter-individual variability in microbiome composition, microbial function, and metabolic profiles in changing gut microbiome community type

**Figure 6.**
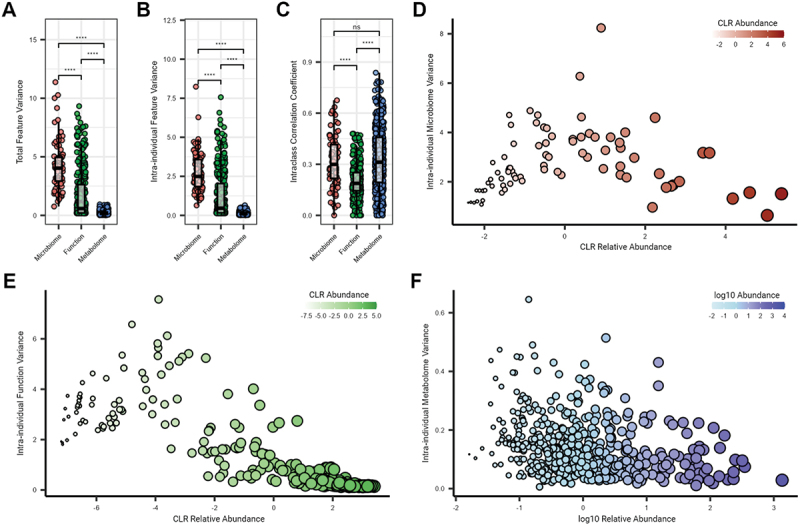
Variability and stability in microbiome composition, microbial function, and metabolic profiles among participants with gut microbiome community type transitions. Boxplots display (A) total variance (combining intra- and inter-individual variability), (B) intra-individual variance, and (C) intraclass correlation coefficients (ICCs) for microbiome features at the genus level, estimated microbial functions, and fecal metabolites (pairwise comparison, ****P_adj_ < 5.27e-08). Boxplot elements denote the median (central line) and interquartile range (box), while the whiskers represent the range within 1.5 times the interquartile distance from the box edges. Points represent the respective features. Scatter plots display intra-individual variability on the y-axis for (D) taxa at the genus level with centered-log ratio relative abundance on the x-axis, (E) estimated functional pathways with centered-log ratio relative abundance on the x-axis, and (F) fecal metabolites with log10 relative abundance on the x-axis. Points are sized and gradient colored according to their relative abundance.

By examining participants who underwent shifts in community type (*n* = 45), our analysis extended to the exploration of variability and stability within these dynamic microbiomes. We assessed various aspects of variability for microbiome composition, estimated microbial function, and metabolite profiles. Utilizing longitudinal sampling data, total variance (encompassing both intra- and inter-individual variability) was calculated for these three domains. Microbiome features at the genus level exhibited significantly higher total variance compared to estimated function and fecal metabolites (pairwise comparison, *P*_adj_ ≤ 2.64e-11; Figure 6A). Interestingly, while the estimated functions displayed lower total variance than the microbiome features, they had greater total variance compared to the fecal metabolome (*P*_adj_ < 2.2e-16). A similar pattern emerged for intra-individual variance, with significant differences observed across all three comparisons (*P*_adj_ ≤5.27e-08; Figure 6B). The observed low total and intra-individual variances in the fecal metabolome suggest that metabolite levels generally exhibit minimal fluctuations. This stability may be attributed to the body’s tight regulatory mechanisms that maintain metabolic homeostasis, leading to less overall variability in metabolite concentrations. Similarly, the relatively low total and intra-individual variances observed in estimated functions indicate a stability in the microbiome’s functional capabilities over time within individuals, as well as reduced variability between individuals.

In assessing ICCs across taxonomic, GM functional, and metabolic profiles, significant differences were identified between genera and fecal metabolites compared to the estimated function (*P*_adj_ ≤ 2.60e-09; Figure 6C), with function exhibiting the lowest ICC. No significant difference was observed between the genera and fecal metabolites (*P*_adj_ = 0.92). The high ICC coupled with low total and intra-individual variance in the fecal metabolome implies that individuals possess distinct metabolic signatures. These signatures, while unique to each individual, demonstrate considerable stability over time. The relatively low ICC for the estimated function suggests that variability in microbial function within individuals over time is comparable to the variability observed between different individuals. This finding, in contrast to the higher individual specificity observed in metabolite profiles, indicates that microbial functions are more conserved and less individual-specific.

We proceeded to investigate the relationships between intra-individual variance and abundance for each of these three layers among participants experiencing shifts in community types. In the case of microbiome composition at the genus level, a negative correlation was observed between intra-individual variance and abundance (Spearman’s *rho* = −0.27, *p* = 0.016; Figure 6D). As established, more abundant genera tend to have less variability within individuals over time.^45^ This pattern may reflect a stabilizing effect of dominant taxa within the gut ecosystem, where higher abundance is associated with reduced responsiveness to fluctuating environmental or host-related factors. The top 10 genera with the highest intra-individual variance included *Akkermansia*, Ruminococcaceae *CAG-352*, *Lachnospira*, *Haemophilus*, *R. gnavus group*, *Dialister*, *Alistipes*, *Parasutterella*, *Prevotella*, and *Escherichia-Shigella*. Of these, *Akkermansia*, a modulator of neighboring bacteria,^51^ and *Alistipes*, generally associated with GM stability,^52,53^ had the greatest relative abundance in the current sample. Similarly, the estimated microbial functions revealed a significant negative correlation (Spearman’s *rho* = −0.92, *p* = 2.2e-16; Figure 6E). The most variable pathways were from the superclass of generation of precursor metabolites and energy (TCA cycle IV [P105-PWY], aerobic respiration I [PWY-3781], superpathway of glycolysis, pyruvate dehydrogenase, TCA, and glyoxylate bypass [GLYCOLYSIS-TCA-GLYOX-BYPASS], and isopropanol biosynthesis [PWY-6876]). Others included amino acid biosynthesis (L-tryptophan biosynthesis [PWY-6629]), superpathway of chorismate metabolism (ALL-CHORISMATE-PWY; chorismate is the principal common precursor of the aromatic amino acids L-tryptophan, L-tyrosine, and L-phenylalanine,^54^ autotrophic CO2 Fixation (reductive acetyl coenzyme A pathway I [CODH-PWY]), peptidoglycan biosynthesis II (PWY-5265), and lipopolysaccharide biosynthesis (superpathway of (Kdo)2-lipid A biosynthesis [KDO-NAGLIPASYN-PWY]). These results indicate that unstable microbial communities have a high potential for metabolic versatility and adaptability. This flexibility might be a critical adaptive mechanism allowing the microbiome to maintain homeostasis and functional integrity amidst the shifting community types observed in our study population. Particularly, the variability in pathways such as the TCA cycle, aerobic respiration, and glycolysis indicates a dynamic regulation of energy production, which could be reflective of the microbiome’s response to varying nutritional inputs and energy demands.

The fecal metabolome demonstrated the most significant negative correlation between variance and abundance (Spearman’s *rho* = −0.31, *p* = 9.42e-14; Figure 6F). While demonstrating much greater stability, metabolites with the highest variance were mainly organic acids and derivatives (e.g., paracetamol sulfate, creatinine, formiminoglutamic acid, L-theanine, cysteinyl-aspartate, and creatine) and lipids and lipid-like molecules (e.g., methyl stearate, taurolithocholic acid 3-sulfate, and pregnanetriol). The variable levels of these organic acids and derivatives could be reflective of shifts in metabolic processes related to detoxification, energy metabolism, and amino acid catabolism. For instance, variations in paracetamol sulfate may indicate changes in detoxification processes,^55^ while fluctuations in creatinine and creatine could be linked to alterations in energy metabolism. Additionally, the presence of compounds like L-theanine and formiminoglutamic acid suggests potential variability in neurotransmitter synthesis and single-carbon metabolism, respectively. Similarly, the variability observed in lipids and lipid-like molecules, such as methyl stearate, taurolithocholic acid 3-sulfate, and pregnanetriol, points to fluctuations in lipid metabolism, which could be influenced by diet, gut microbial activity, or host physiology. These lipid molecules play crucial roles in cell membrane structure, signaling, and energy storage, and their variability might reflect the gut ecosystem’s response to dietary lipids or shifts in the composition of the GM.

## Discussion

This study provides a comprehensive view of the GM and fecal metabolome in college students during their transition to college life. We identified four distinct GM community types, suggesting a degree of GM maturity in these students, with notable prevalence of *Blautia* and *Bacteroides* 2, the latter often associated with disease.^40–43^ We also found distinct metabolic signatures distinguishing *Prevotella* and *Blautia* dominance from *Bacteroides*. While we observed several ecological features characteristic of an adult GM, we also detected markers reminiscent of younger individuals and identified a degree of plasticity, as evidenced by the number of community-type shifts observed. Nearly half of the participants in our longitudinal cohort experienced GM community shifts during their first year of college, highlighting the GM’s taxonomic adaptability. These transitions were associated with significant changes in microbial composition and diversity, with individual-specific influences playing a major role. While diet and behavioral factors influenced GM composition to some extent, it did not independently predict community transitions within the study’s timeframe.

Our initial objective was to evaluate the microbial and metabolomic signatures of the GM within this diverse group of emerging adults during their college years. Through our analysis, we identified four distinct community types primarily driven by the presence of *Bacteroides* (Bact1 and Bact2), *Blautia* (Blau), and *Prevotella* (Prev). These community types exhibited similarities to those observed in adults,^56–60^ suggesting a mature GM configuration among these college students. Of particular significance was the prevalence of *Blautia* within one of the community types, previously found to be an important contributor of *Ruminococcus* enterotyping (both from the order Clostridiales).^56^ The presence of *Blautia* in the GM has been associated with specific metabolic functions and potential implications for host health, including the expression of probiotic characteristics^61^ and butyrate and acetate production.^62^ Though it has also been associated with visceral fat accumulation in large population-based studies.^63^ Additionally, we observed the presence of Bact2, a community type often regarded as disease-associated based on previous research.^40–43,64^ Remarkably, both the Bact2 and Prev communities displayed higher alpha diversity, indicating a more diverse array of microbial features compared to prior studies. This finding contrasts with existing research in relation to Bact2,^42,43,64^ which has suggested reduced diversity in certain disease-associated GM configurations. These results suggest that the GM of college students in our study exhibited unique characteristics and responses that may be influenced by their specific lifestyle and environmental factors.

We uncovered distinct metabolic signatures differentiating both *Prevotella* and *Blautia* dominated states from *Bacteroides* dominance. The metabolic distinction was most pronounced in the *Prevotella*-to-*Bacteroides* (P/B) comparison. We observed positive correlations between the P/B ratio and metabolites such as octadecanamide, calcium gluceptate, trans-2-dodecenoylcarnitine, and diaminopimelic acid. These metabolites play diverse roles, including involvement in lipid metabolism,^65^ amino acid metabolism,^66^ and formation of cell wall peptidoglycans.^67^ The positive correlations with the P/B ratio suggest potential metabolic shifts in individuals with higher *Prevotella* dominance, possibly reflecting alterations in nutrient metabolism and immune response. Conversely, we found negative correlations between the P/B ratio and metabolites, such as homocitrulline, 1-methyluric acid, p-cresol sulfate, 8-hydroxy-7-methylguanine, and cholesterol sulfate. P-cresol sulfate, for instance, is produced by gut bacteria during the metabolism of tyrosine and phenylalanine from dietary protein and is known as a protein-bound uremic toxin.^68–70^ The negative correlation with the P/B ratio suggests that individuals with higher *Bacteroides* dominance may exhibit elevated levels of this potentially harmful metabolite. Similarly, elevated levels of homocitrulline may indicate alterations in amino acid metabolism, particularly in the urea cycle.^71^ The presence of cholesterol sulfate in fecal metabolites suggests that gut bacteria may be involved in the metabolism of cholesterol and related compounds. The presence of 8-hydroxy-7-methylguanine, a modified nucleoside associated with red meat intake, may indicate oxidative stress and DNA damage processes within the gut environment.^72^ On the other hand, kynurenic acid, a metabolite produced during the breakdown of tryptophan with potential neuroprotective and anti-inflammatory properties,^73,74^ showed a positive correlation with the P/B ratio. This finding suggests that individuals with a higher *Prevotella* dominance may have a metabolite profile associated with potential neuroprotective effects and reduced inflammation.

We also conducted a similar analysis for the *Blautia*-to-*Bacteroides* (B/B) ratio, identifying six significant metabolites. However, the correlations were generally smaller compared to the P/B ratio, suggesting that the metabolic differences associated with the B/B ratio may be less pronounced. The analysis of the B/B ratio revealed correlations with metabolites representing various metabolic pathways, including amino acid metabolism (allysine and L-gamma-glutamyl-L-leucine), bile acid metabolism (lithocholic acid), and antioxidant processes (dehydroascorbic acid). Similar to the P/B ratio, the B/B ratio also exhibited a negative correlation with p-cresol sulfate, suggesting that individuals with higher Blautia dominance may have lower levels of p-cresol sulfate, potentially indicating differences in protein metabolism by their gut microbiota.

The second primary objective of this study was to investigate shifts in GM composition during the first year of college. Importantly, sample collection covered seasonal variation via the beginning (Fall semester), middle (end of Fall, beginning of Spring Semesters), and end (Spring semester) of the academic year. Our longitudinal analysis of a subset of participants who provided complete sample sets revealed key taxa associated with intra-individual GM changes. *Parasutterella* and *R. gnavus group* showed positive associations with Bray-Curtis dissimilarity, indicating their potential roles in driving compositional variability. In contrast, genera associated with butyrate production, such as *Faecalibacterium* and *E. ventriosum group*, were negatively correlated with intra-individual variability, suggesting their stabilizing effects on the GM. Of interest, *Faecalibacterium* has previously been identified as a stabilizing factor in longitudinal studies of healthy adults.^45^ It is a taxon that typically declines with age but is associated with healthy aging.^75–78^ *Eubacterium ventriosum* has been observed to decrease in centenarians^79^ and is proposed as a biomarker for low risk of colorectal cancer, showing significant enrichment in healthy individuals compared to those with colorectal cancer in diverse populations.^80^ Similarly, *Parasutterella* has been found to be more abundant in the GM of young healthy (20–39 years old) compared to older individuals.^81^ This taxon is positively correlated with acetate production^82^ and is a significant driver of butyrate production by members of the *Faecalibacterium* genus through a process known as acetate-cross feeding in the healthy human gut.^83^ Indeed, supplementing the culture medium with acetate (33–50 mM) stimulates *Faecalibacterium* growth.^84–86^

Notably, *R. gnavus group* exhibited markedly lower abundances in individuals with Bact1 and Prev community types in comparison to those transitioning to alternative community types. *Ruminococcus gnavus group* is recognized as a pathobiont, showing increased prevalence with age, particularly in the context of unhealthy aging.^5,87,88^ Transient blooms of *R. gnavus* appear to be associated with inflammatory states like Crohn’s disease.^89,90^ This taxon has the ability to forage host mucin,^91^ potentially heightening exposure to innate immune cells in the gut wall and exacerbating inflammatory responses.^92^ Its prevalence is associated with diets rich in animal products^87^ but can be mitigated through targeted approaches such as the consumption of polyphenol-rich foods,^93^ generally associated with a *Prevotella*-dominate configuration. Indeed, our investigation revealed that *R. gnavus group* was significantly less abundant in individuals with Bact1 and Prev community types, including Blau, which is linked to higher dietary protein intake.^94^ The Blautia-dominated community stood out for its significantly lower Shannon diversity index, indicating a more homogenous microbial composition within this group. This observation suggests that certain community types may be more resilient or stable than others, and this resilience could be influenced by various factors.

Our investigation into the dynamics of GM community types over the academic year unveiled intriguing patterns of transition and stability within the microbiome. Nearly half of the participants experienced shifts in their community states during the academic year, highlighting the adaptability of the GM to changing conditions and environmental factors. Comparing individuals with stable community types to those undergoing transitions, we observed significant differences in microbial composition and diversity. Specifically, participants transitioning between community types exhibited higher microbial variability and lower alpha diversity, indicating that these transitions entail substantial changes in the gut microbiota. Community types are typically associated with long-term dietary patterns, such as protein and animal fat (*Bacteroides*) versus carbohydrates (*Prevotella*), and are considered stable over shorter time frames.^95^ However, Vandeputte et al.. (2021) recently reported that normal day-to-day events are sufficient to trigger transient community-type switches.^40^ Moreover, fluctuations in protein, carbohydrate/fiber, and sodium intake also seem to affect microbiome profiles over time. While diet and behavioral attributes had a lesser impact on these transitions in our study, inter-individual variability emerged as the predominant source of variation in community composition, underscoring the strong influence of individual-specific factors on the GM. Although diet-related variables, such as red/processed meat consumption, dietary fiber intake, and fruit/vegetable consumption contributed to some of the observed variance, they did not independently associate with the probability of switching between community types within the study’s timeframe. This suggests that while diet may influence GM composition, it is not the sole determinant of community transitions. Finally, regarding other important factors such as seasonal variation and sex, we noted minimal influence. Previous studies have reported GM compositional shifts over different seasons in rural^96,97^ and hunter-gatherer populations.^98,99^ Our results may be influenced by the location and level of modern urban development (e.g., climate control and constancy of food supply), though our less longitudinally dense sampling was a limitation. The minimal influence of sex in our study, despite having both female and male participants, is notable given that steroidal hormones play important roles in adaptation to environmental changes during late-stage adolescence.^100^ Overall, more research is needed to comprehensively explore the intricate interplay between lifestyle factors in this population and their potential cascading effects on GM structure. This will enhance our understanding of the variables that shape the microbial landscape during the critical transition to adult life.

We further explored variability and stability within dynamic microbiomes among participants who experienced shifts in community types. We observed that microbiome features at the genus level exhibited significantly higher total variance compared to estimated function and fecal metabolites. Among the top 10 genera with the highest intra-individual variance, *Akkermansia* and *Alistipes* were notable, as were *Parasutterella* and *R. gnavus group*, the latter two aligning with our previous assessment of genera stability for the entire cohort. *Alistipes* has previously been shown to have relatively low variability over longer periods of time.^53,101^ Despite this taxon’s reported resilience, its 13 species have varying susceptibility and tolerance to bile acids,^102^ which may reflect changes in dietary patterns not captured in our study. Indeed, *Alistipes* has been associated with higher fat diets and animal-protein-rich diets^103^ and can increase rapidly upon switching to a high fat intake.^104^ *Akkermansia* also has been found to be elevated after high fat diets in mice,^105^ though is generally regarded to be associated with metabolic improvement in humans.^106^ These microbes may possess a degree of adaptability and could bloom depending on substrate availability from the host and changing landscapes within the GM. Relatedly, the most variable pathways included those related to energy production, amino acid biosynthesis, chorismate metabolism, autotrophic CO2 fixation, and peptidoglycan biosynthesis, suggesting a dynamic regulation of energy production and metabolic adaptability in unstable microbial communities. The fecal metabolome demonstrated the lowest total variance, indicating that metabolite levels exhibit minimal fluctuation. This stability may be attributed to the body’s regulatory mechanisms that maintain metabolic homeostasis, leading to less overall variability in metabolite concentrations. Metabolites with the highest variance were mainly organic acids and derivatives and lipids and lipid-like molecules. The variability in metabolite levels may indicate changes in detoxification processes, energy metabolism, amino acid catabolism, neurotransmitter synthesis, and lipid metabolism within the gut ecosystem. The observed stability in the fecal metabolome suggests that metabolite concentrations are tightly regulated and maintained within a relatively narrow range.

This study had several strengths, including a diverse and well-characterized cohort of college students, extensive health and behavior data, and comprehensive profiling of both the microbiome and metabolome. However, it also had limitations, such as limited dietary data captured by questionnaires, which may have affected our ability to fully elucidate the relationships between diet and the GM. Additionally, other behavioral factors such as depression and anxiety, which are prevalent in this stress-prone population due to various factors (e.g., academic pressure^107^ and food insecurity,^108^ deserve more in-depth analysis. Although our analyses yielded few significant associations with these factors, they remain important areas for future research. Moreover, longitudinal assessment was limited to three samples. However, previous work has shown that collecting minimally three longitudinal samples allows for calculating equilibrium abundances with substantially higher accuracy, as well as estimating temporal variation with a minimum number of samples.^40^ Lastly, data were obtained from 16S rRNA sequencing, which may not allow in-depth interpretation of the results based on taxonomic resolution.

In conclusion, this study provides a comprehensive view of the GM and fecal metabolome dynamics in college students during their transition to college life. Four distinct GM community types were identified, suggesting GM maturity in these students, with notable prevalence of Blau and Bact2. Additionally, we found unique metabolic signatures distinguishing *Prevotella* and *Blautia* dominance from *Bacteroides*. Almost half of the participants experienced GM community shifts during their first year of college, highlighting the GM’s adaptability, with these transitions associated with significant changes in microbial composition and diversity. While diet and behavior had some influence, individual-specific factors played a major role. Key genera, such as *Alistipes*, *Akkermansia*, *Parasutterella*, and the *R. gnavus group*, showed adaptability and variability within dynamic microbiomes. Our findings also revealed significant correlations between GM community types and fecal metabolites, providing insights into potential metabolic shifts. Despite some limitations, our study contributes to a deeper understanding of GM dynamics in emerging adults, emphasizing the intricate interplay between the GM, metabolism, and individual-specific factors, with implications for overall health and well-being. Further research can explore the long-term consequences of these findings.

## Supplementary Material

Gut Microbes Rep_SI_v2_061024_CW_072624.docx

## Data Availability

Due to the vulnerable nature of adolescent and emerging adult populations, researchers did not seek participant consent to share study data. Therefore, participants of this study did not agree for their data to be shared publicly, and supporting data are not available. However, we have provided all relevant QIIME2 artifacts and scripts used in the quality control and data analysis of this study in a GitHub repository to enhance transparency and reproducibility of our methods. These artifacts include demultiplexed sequence quality visualizations, denoising statistics, merged feature table summaries, and merged representative sequence summaries. The repository can be accessed at https://github.com/Alex-E-Mohr/devilWASTE.
