# Supplementary material for: Microbial Ecology and Metabolism of Emerging Adulthood: Gut Microbiome Insights from a College Freshman Cohort
Source: Gut Microbes Rep. 2024 Aug 19;1(1):2387936. doi: 10.1080/29933935.2024.2387936 (PMC11361303; doi:10.1080/29933935.2024.2387936)
Supplement: Gut Microbes Rep_SI_v2_061024_CW_072624.docx [file KGMR_A_2387936_SM8140.docx]

**Supplemental Information**

**Microbial Ecology and Metabolism of Emerging Adulthood: Gut Microbiome Insights from a College Freshman Cohort**

Alex E. Mohr^1,2^, Paniz Jasbi^1,3^, Irene van Woerden^4^, Jinhua Chi^1^, Haiwei Gu^1^, Meg Bruening^1,5^, Corrie M. Whisner^1,2^*

^1^College of Health Solutions, Arizona State University, Phoenix, AZ, USA

^2^Center for Health Through Microbiomes, Biodesign Institute, Arizona State University, Tempe, AZ, USA

^3^Biodesign Center for Personalized Diagnostics, School of Molecular Sciences, Arizona State University, Tempe, AZ USA

^4^Community and Public Health, Idaho State University, Pocatello, ID, USA

^5^Department of Nutritional Sciences, College of Health and Human Development, Pennsylvania State University, University Park, PA, USA

*Corresponding AuthorEmail:

[cwhisner@asu.edu](mailto:cwhisner@asu.edu)

**Page S-2, Table S1.** Participant demographics at first fecal sample collection.

**Page S-3, Table S2.** Adonis model parameters and term results for Bray-Curtis dissimilarity matrix of devilWASTE samples (*n* = 485).

**Page S-4, Table S3.** Multiple regression model parameters and term results for Prevotella/Bacteroides ratio of devilWASTE samples (*n* = 485).

**Page S-5, Table S4.** Multiple regression model parameters and term results for Blautia/Bacteroides ratio of devilWASTE samples (*n* = 485).

**Page S-6, Table S5.** Input parameters and term results for Chi square test comparing observed Dirichlet multinomial mixture community type transitions to expected transition rates of longitudinal devilWASTE samples (*n* = 279).

**Page S-7, Figure S1.** (A) Goodness-of-fit assessed for number of Dirichlet components via Laplace approximation. Four components were selected and used for further analysis. The main community drivers (≥99% contribution) of (B) Type 1 (dominated by Bacteroides), (C) Type 2 (dominated by Bacteroides), (D) Type 3 (dominated by Blautia), and (E) Type 4 (dominated by Prevotella).

**Page S-8, Figure S2.** Metabolic signatures associated with *Blautia*/*Bacteroides* ratio and correlation analysis. (A) Fecal metabolites, categorized by super-family, with corresponding -log10 p-values derived from regression models predicting *Blautia/Bacteroides* ratio while adjusting for sex, BMI, and time. Metabolites above the light red line are deemed significant after multiple-hypothesis correction (*P*_adj_ < 0.05), while the blue line represents the unadjusted *P*-value threshold. (B) Spearman correlation coefficients illustrating the relationships between each metabolite and the *Blautia/Bacteroides* ratio, after controlling for covariates and applying multiple-hypothesis correction (*P*_adj_ < 0.05).

| **Table S1.** Participant demographics at first fecal sample collection. | | | | |
| --- | --- | --- | --- | --- |
| Characteristic | Total (*n* = 246) | Females (*n* = 160) | Males (*n* = 86) | *P*-value |
| Age (years) | 18.59 ± 0.72 | 18.58 ± 0.76 | 18.61 ± 0.63 | 0.788 |
| Race/ethnicity % (*n*)  White  Black  Hispanic  Other | 43.9 (108)  12.6 (31)  26.4 (65)  17.1 (42) | 43.1 (69)  15.6 (25)  25.6 (41)  15.6 (25) | 45.3 (39)  7.0 (6)  27.9 (24)  19.8 (17) |  |
| Height (cm) | 168.50 ± 10.36 | 163.24 ± 7.39 | 178.29 ± 7.63 | < 0.001 |
| Weight (kg) | 70.33 ± 16.76 | 65.19 ± 15.65 | 79.89 ± 14.46 | < 0.001 |
| Waist circumference (cm) | 82.07 ± 12.55 | 80.09 ± 12.96 | 85.76 ± 10.89 | < 0.001 |
| Waist/hip ratio | 0.82 ± 0.07 | 0.81 ± 0.07 | 0.85 ± 0.06 | < 0.001 |
| BMI (kg/m^2^) | 24.64 ± 4.88 | 24.39 ± 5.25 | 25.09 ± 4.09 | 0.253 |
| Moderate to vigorous physical activity (min) | 43.80 ± 30.93 | 37.73 ± 28.89 | 55.09 ± 31.59 | < 0.001 |
| Depression score | 2.04 ± 0.78 | 2.07 ± 0.73 | 1.98 ± 0.87 | 0.840 |
| Anxiety score | 1.97 ± 1.01 | 2.06 ± 1.01 | 1.79 ± 0.99 | 0.203 |
| Hours of nightly sleep^a^ | 7.24 ± 1.18 | 7.31 ± 1.11 | 7.10 ± 1.29 | 0.198 |
| Alcohol intake^b^ | 3.39 ± 6.02 | 2.94 ± 4.34 | 4.43 ± 6.64 | 0.149 |
| Fruit/vegetable intake^c^ | 2.45 ± 1.05 | 2.26 ± 0.78 | 2.80 ± 1.38 | < 0.001 |
| Whole grain intake^c^ | 0.79 ± 0.41 | 0.74 ± 0.34 | 0.88 ± 0.49 | 0.028 |
| Dairy intake^c^ | 2.04 ± 1.21 | 1.67 ± 0.67 | 2.72 ± 1.62 | < 0.001 |
| Red/processed meat  intake^d^ | 0.85 ± 0.84 | 0.64 ± 0.60 | 1.24 ± 1.05 | < 0.001 |
| Daily sugar intake (g) | 20.49 ± 14.87 | 17.30 ± 7.12 | 26.43 ± 22.09 | < 0.001 |
| Daily fiber intake (g) | 16.29 ± 4.72 | 14.82 ± 3.39 | 19.02 ± 5.59 | < 0.001 |
| Data displayed as mean ± SD, unless stated otherwise.  Superscripts: ^a^Average of combined weekday and weekend nightly sleep hours; ^b^Expressed as number of beverages over the last 7 days; ^c^Expressed as daily cup equivalents; ^d^Daily number of servings. | | | | |

| **Table S2.** Adonis model parameters and term results for Bray-Curtis dissimilarity matrix of devilWASTE samples (*n* = 485). | | | | | |
| --- | --- | --- | --- | --- | --- |
|  | **DF** | **SS** | ***R*^2^** | **F** | **p** |
| **as.character(Participant)** | 241 | 86.314 | 0.645 | 2.428 | < 0.001 |
| **DMM Assignment** | 3 | 10.006 | 0.075 | 22.658 | < 0.001 |
| **Sex** | 1 | 0.503 | 0.004 | 3.419 | < 0.001 |
| **Race/ethnicity** | 3 | 1.617 | 0.012 | 3.661 | < 0.001 |
| **BMI** | 1 | 0.489 | 0.004 | 3.321 | < 0.001 |
| **Time of collection** | 1 | 0.391 | 0.003 | 3.321 | < 0.001 |
| **Residuals** | 234 | 34.445 | 0.258 |  |  |
| **Total** | 484 | 133.585 | 1.000 |  |  |
| Abbreviations: DF, degrees of freedom; SS, sum of squares. | | | | | |

| **Table S3.** Multiple regression model parameters and term results for *Prevotella/Bacteroides* ratio of devilWASTE samples (*n* = 485). | | | | | | | |
| --- | --- | --- | --- | --- | --- | --- | --- |
|  | **DF** | **SS** | **MS** | **F** | ***P*** | ***P*_adj_** | **%Exp** |
| **Time of collection** | 1 | 2.965 | 2.965 | 0.489 | 0.485 | 1.000 | 0.079 |
| **Campus location** | 1 | 4.782 | 4.782 | 0.789 | 0.375 | 1.000 | 0.128 |
| **Dormitory** | 1 | 82.476 | 82.476 | 13.612 | 2.8e-04 | 6.2e-03 | 2.204 |
| **Credits** | 1 | 60.446 | 60.446 | 9.965 | 0.002 | 0.039 | 1.616 |
| **GPA** | 1 | 111.434 | 111.434 | 18.371 | 2.6e-05 | 0.001 | 2.978 |
| **Sex** | 1 | 7.973 | 7.973 | 1.314 | 0.253 | 1.000 | 0.213 |
| **Race/ethnicity** | 3 | 36.813 | 12.271 | 2.023 | 0.111 | 1.000 | 0.984 |
| **Age** | 1 | 3.853 | 3.853 | 0.635 | 0.426 | 1.000 | 0.103 |
| **MVPA** | 1 | 2.383 | 2.383 | 0.393 | 0.531 | 1.000 | 0.064 |
| **Depression score** | 1 | 31.264 | 31.264 | 5.154 | 0.024 | 0.531 | 0.835 |
| **Anxiety score** | 1 | 17.829 | 17.829 | 2.939 | 0.088 | 1.000 | 0.477 |
| **BMI** | 1 | 0.202 | 0.202 | 0.033 | 0.885 | 1.000 | 0.005 |
| **WHR** | 1 | 17.195 | 17.195 | 2.835 | 0.094 | 1.000 | 0.459 |
| **Hours of nightly sleep** | 1 | 51.434 | 51.434 | 8.488 | 0.004 | 0.088 | 1.375 |
| **Alcohol intake** | 1 | 5.8e-04 | 5.8e-04 | 9.6e-05 | 0.992 | 1.000 | 1.6e-05 |
| **Fruit/vegetable intake** | 1 | 9.470 | 9.470 | 1.561 | 0.213 | 1.000 | 0.253 |
| **Whole grain intake** | 1 | 0.083 | 0.083 | 0.014 | 0.906 | 1.000 | 0.002 |
| **Dairy intake** | 1 | 0.853 | 0.853 | 0.141 | 0.708 | 1.000 | 0.023 |
| **Red/processed meat intake** | 1 | 23.987 | 23.987 | 3.959 | 0.047 | 1.000 | 0.641 |
| **Daily sugar intake** | 1 | 30.598 | 30.598 | 5.044 | 0.026 | 0.564 | 0.818 |
| **Daily fiber intake** | 1 | 22.744 | 22.744 | 3.749 | 0.054 | 1.000 | 0.608 |
| **DMM assignment** | 2 | 1827.573 | 913.786 | 1.506 | 1.5e-42 | 3.4e-10 | 48.846 |
| **Residuals** | 230 | 1395.115 | 6.066 |  |  |  |  |
| Abbreviations: DF, degrees of freedom; SS, sum of squares; MS, mean square; %Exp, percent of explained variance. | | | | | | | |

| **Table S4.** Multiple regression model parameters and term results for *Blautia/Bacteroides* ratio of devilWASTE samples (*n* = 485). | | | | | | | |
| --- | --- | --- | --- | --- | --- | --- | --- |
|  | **DF** | **SS** | **MS** | **F** | ***P*** | ***P*_adj_** | **%Exp** |
| **Time of collection** | 1 | 3.292 | 3.292 | 4.179 | 0.042 | 0.917 | 1.357 |
| **Campus location** | 1 | 0.555 | 0.555 | 0.705 | 0.401 | 1.000 | 0.229 |
| **Dormitory** | 1 | 2.803 | 2.803 | 3.571 | 0.060 | 1.000 | 1.156 |
| **Credits** | 1 | 3.528 | 3.528 | 4.494 | 0.035 | 0.772 | 1.455 |
| **GPA** | 1 | 0.359 | 0.359 | 0.458 | 0.499 | 1.000 | 0.148 |
| **Sex** | 1 | 0.173 | 0.173 | 0.220 | 0.639 | 1.000 | 0.071 |
| **Race/ethnicity** | 3 | 6.384 | 2.128 | 2.711 | 0.046 | 1.000 | 2.632 |
| **Age** | 1 | 0.019 | 0.019 | 0.024 | 0.876 | 1.000 | 0.008 |
| **MVPA** | 1 | 0.173 | 0.173 | 0.221 | 0.639 | 1.000 | 0.071 |
| **BMI** | 1 | 0.466 | 0.466 | 0.594 | 0.446 | 1.000 | 0.192 |
| **Depression score** | 1 | 0.254 | 0.254 | 0.324 | 0.569 | 1.000 | 0.105 |
| **Anxiety score** | 1 | 2.416 | 2.416 | 3.077 | 0.081 | 1.000 | 0.996 |
| **WHR** | 1 | 3.363 | 3.363 | 4.284 | 0.039 | 0.871 | 1.387 |
| **Hours of nightly sleep** | 1 | 0.061 | 0.061 | 0.077 | 0.782 | 1.000 | 0.025 |
| **Alcohol intake** | 1 | 0.717 | 0.717 | 0.913 | 0.341 | 1.000 | 0.295 |
| **Fruit/vegetable intake** | 1 | 0.933 | 0.933 | 1.189 | 0.277 | 1.000 | 0.385 |
| **Whole grain intake** | 1 | 1.140 | 1.140 | 1.453 | 0.229 | 1.000 | 0.470 |
| **Dairy intake** | 1 | 0.033 | 0.033 | 0.042 | 0.837 | 1.000 | 0.014 |
| **Red/processed meat intake** | 1 | 0.021 | 0.021 | 0.027 | 0.870 | 1.000 | 0.009 |
| **Daily sugar intake** | 1 | 0.013 | 0.013 | 0.002 | 0.898 | 1.000 | 0.005 |
| **Daily fiber intake** | 1 | 1.727 | 1.727 | 2.199 | 0.139 | 1.000 | 0.712 |
| **DMM assignment** | 2 | 33.546 | 16.773 | 21.368 | 3.1e-09 | 6.8e-08 | 13.832 |
| **Residuals** | 230 | 180.539 | 0.785 |  |  |  |  |
| Abbreviations: DF, degrees of freedom; SS, sum of squares; MS, mean square; %Exp, percent of explained variance. | | | | | | | |

| **Table S5.** Input parameters and term results for Chi square test comparing observed Dirichlet multinomial mixture community type transitions to expected transition rates of longitudinal devilWASTE samples (*n* = 279). | | | | |
| --- | --- | --- | --- | --- |
| **Transition** | **Observed** | **Expected** | **Residuals** | **STD Residuals** |
| Bact1-Bact1 | 52 | 0.369 | -16.651 | -2.009 |
| Bact1-Bact2 | 11 | 0.222 | -30.312 | -4.716 |
| Bact1-Blau | 8 | 0.206 | -30.274 | -4.893 |
| Bact1-Prev | 2 | 0.114 | -19.263 | -4.178 |
| Bact2-Bact1 | 10 | 0.222 | -31.312 | -4.872 |
| Bact2-Bact2 | 32 | 0.134 | 7.139 | 1.432 |
| Bact2-Blau | 2 | 0.124 | -21.032 | -4.382 |
| Bact2-Prev | 3 | 0.069 | -9.796 | -2.738 |
| Blau-Bact1 | 10 | 0.206 | -28.274 | -4.570 |
| Blau-Bact2 | 2 | 0.124 | -21.032 | -4.382 |
| Blau-Blau | 29 | 0.115 | 7.661 | 1.659 |
| Blau-Prev | 1 | 0.064 | -10.855 | -3.153 |
| Prev-Bact1 | 2 | 0.114 | -19.263 | -4.178 |
| Prev-Bact2 | 3 | 0.069 | -9.796 | -2.738 |
| Prev-Blau | 2 | 0.064 | -9.855 | -2.862 |
| Prev-Prev | 17 | 0.035 | 10.414 | 4.058 |
| Abbreviations: Bact1, Bacteroides-1; Bact2, Bacteroides-2; Blau, Blautia; Prev, Prevotella; STD, standardized. | | | | |


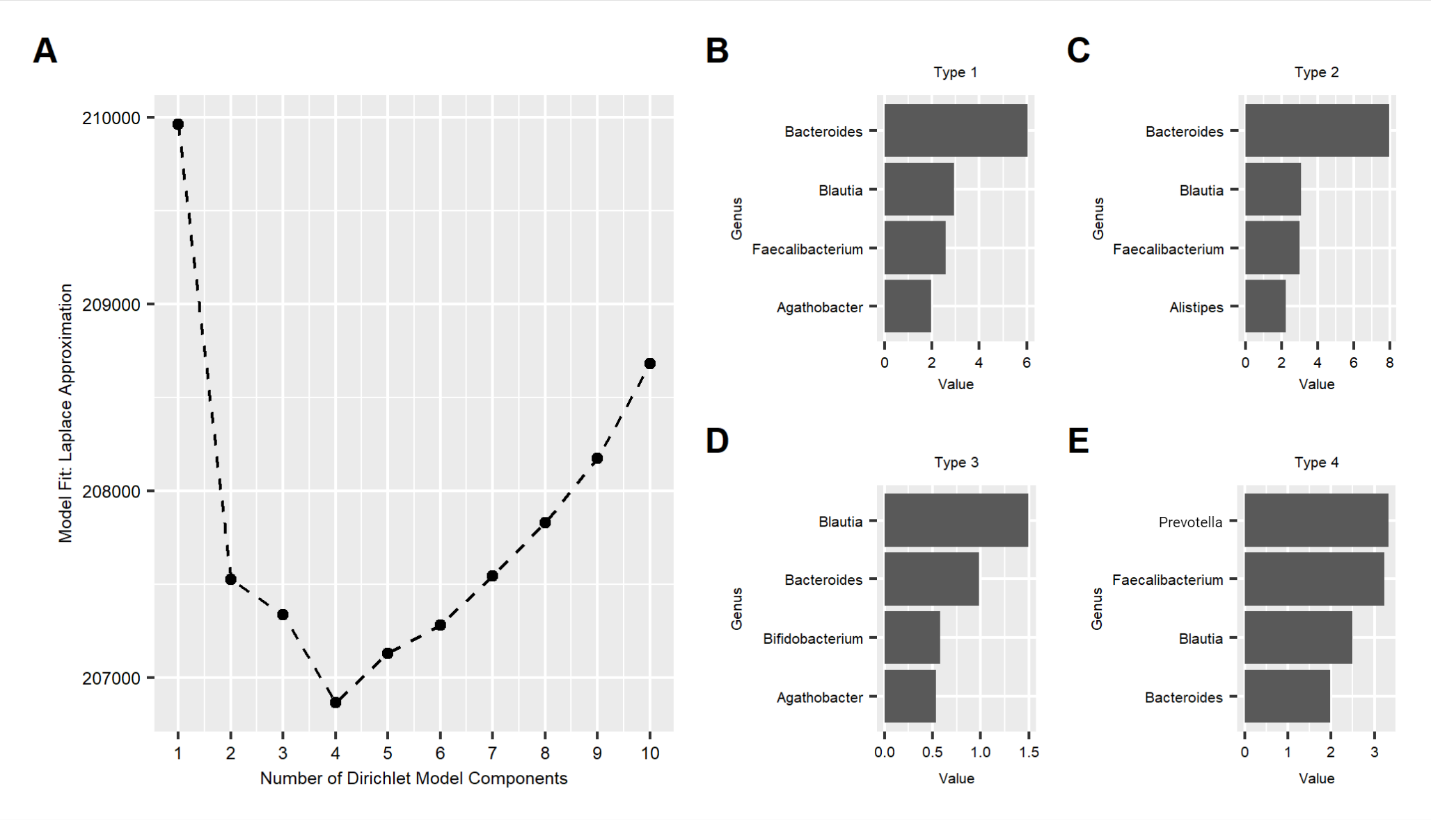


**Figure S1.** (**A**) Goodness-of-fit assessed for number of Dirichlet components via Laplace approximation. Four components were selected and used for further analysis. The main community drivers (≥99% contribution) of (**B**) Type 1 (dominated by Bacteroides), (**C**) Type 2 (dominated by Bacteroides), (**D**) Type 3 (dominated by Blautia), and (**E**) Type 4 (dominated by Prevotella).


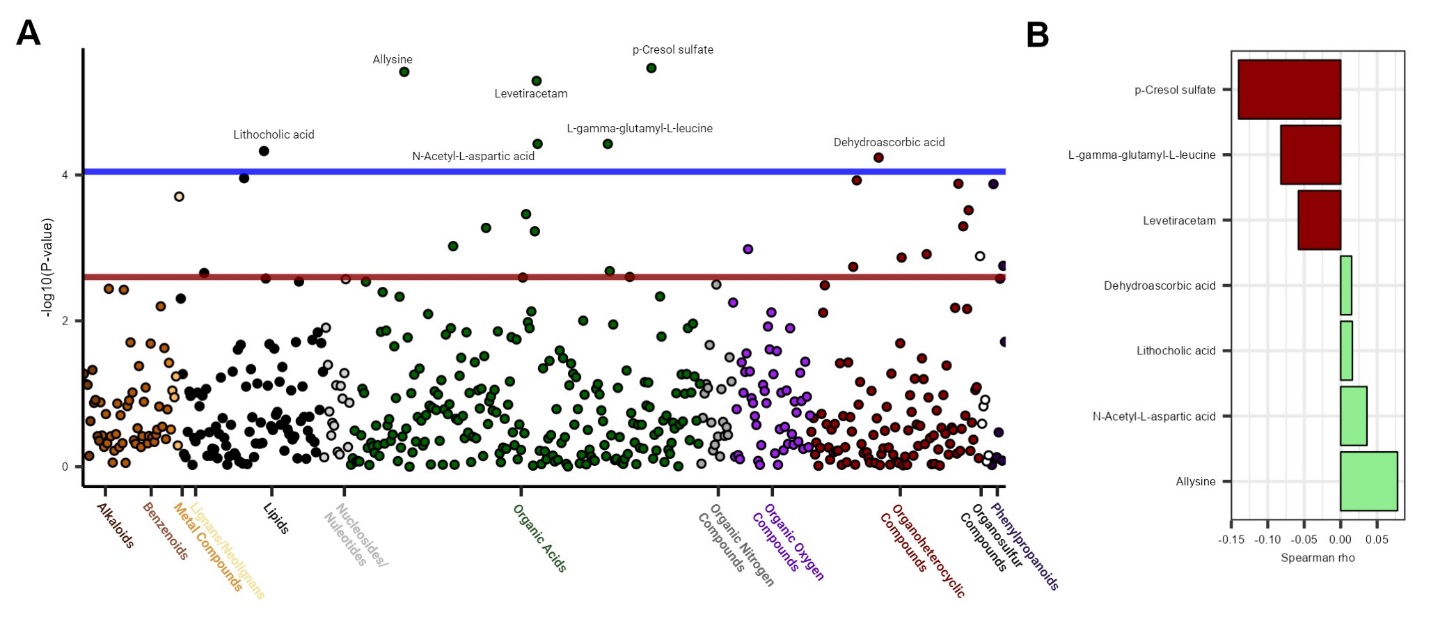


**Figure S2.** Metabolic signatures associated with *Blautia/Bacteroides* ratio and correlation analysis. (**A**) Fecal metabolites, categorized by super-family, with corresponding -log10 p-values derived from regression models predicting *Blautia*/*Bacteroides* ratio while adjusting for sex, BMI, and time. Metabolites above the light red line are deemed significant after multiple-hypothesis correction (*P*_adj_ < 0.05), while the blue line represents the unadjusted *P*-value threshold. (**B**) Spearman correlation coefficients illustrating the relationships between each metabolite and the *Blautia*/*Bacteroides* ratio, after controlling for covariates and applying multiple-hypothesis correction (*P*_adj_ < 0.05).
